# Siloxane-Modified UV-Curable Castor-Oil-Based Waterborne Polyurethane Superhydrophobic Coatings

**DOI:** 10.3390/polym15234588

**Published:** 2023-11-30

**Authors:** Qianhui Yu, Zengshuai Zhang, Pengyun Tan, Jiahao Zhou, Xiaojing Ma, Yingqing Shao, Shuangying Wei, Zhenhua Gao

**Affiliations:** 1 Engineering Research Center of Advanced Wooden Materials, Northeast Forestry University, Ministry of Education, Harbin 150040, China; 15121877581@163.com (Q.Y.); 15735413699@163.com (Z.Z.); dlcltpy@nefu.edu.cn (P.T.); 18714348152@163.com (J.Z.); maxiao.jing@163.com (X.M.); 2022111431@nefu.edu.cn (Y.S.); 2 College of Materials Science and Engineering, Northeast Forestry University, Harbin 150040, China; 3 Key Laboratory of Bio-based Material Science and Technology, Northeast Forestry University, Harbin 150040, China

**Keywords:** superhydrophobic coating, self-cleaning, castor oil, silicone modification, UV curing, waterborne polyurethane coating

## Abstract

In recent years, superhydrophobic coatings with self-cleaning abilities have attracted considerable attention. In this study, we introduced hydroxyl-terminated polydimethylsiloxane (OH−PDMS) into castor-oil-based waterborne polyurethanes and synthesized silicone-modified castor-oil-based UV-curable waterborne polyurethanes (SCWPU). Further, we identified the optimal amount of OH−PDMS to be added and introduced different amounts of micro- and nanoscale heptadecafluorodecyltrimethoxysilane-modified SiO_2_ particles (FAS−SiO_2_) to prepare rough-surface SCWPU coatings with dense micro- and nanostructures, thus realizing waterborne superhydrophobic coatings. The results show that when the OH−PDMS content was 11 wt% and the total addition of FAS−SiO_2_ particles was 50% (with a 1:1:1 ratio of 100 nm, 1 µm, and 10 nm particles), the coatings exhibited a self-cleaning ability and superhydrophobicity with a contact angle of (152.36 ± 2.29)° and a roll-off angle of (4.9 ± 1.0)°. This castor-oil-based waterborne superhydrophobic coating has great potential for waterproofing, anti-fouling, anti-corrosion, and other applications.

## 1. Introduction

Superhydrophobic coatings are a special class of coatings whose properties, like those of the lotus leaf [1], prevent water droplets from spreading to wet the surface of an object and instead ensure that the droplets roll freely off the surface. Therefore, superhydrophobic coatings have a wide range of potential applications, such as in self-cleaning, anti-icing, and anti-corrosion applications [2,3]. Furthermore, superhydrophobic coatings provide a new solution to address the deficiencies of waterborne coatings in terms of water resistance [4]. In addition, by endowing waterborne coatings with superhydrophobicity, their hydrophobicity can be improved while still maintaining their environmental friendliness [5,6,7].

The methods for constructing superhydrophobic surfaces include the sol-gel method, the plasma etching method, the nanoparticle self-assembly method, the microphase separation method, the template method, and the soft microfilming technique [8,9,10,11]. As one among the many materials used for preparing superhydrophobic coatings, polysiloxanes have good hydrophobicity and weatherability, and when introduced into waterborne polyurethanes, the resulting coatings contain low-surface-energy Si elements, which have low surface tension at room temperature and can achieve hydrophobic conditions [12]. As a result, polysiloxanes have been widely used for preparing hydrophobic coatings in recent years [13,14]. Conventional waterborne polyurethanes face the problem of low water resistance (water contact angle is generally below 90°) and the water resistance of waterborne polyurethanes modified by OH−PDMS can be improved [15]. However, the hydrophobic performance of such coatings remains inadequate, and the contact angle does not exceed 110° [16,17,18]. In order to further enhance the hydrophobic performance of coatings, some researchers have incorporated modified nanoparticles, such as TiO_2_, ZnO, and SiO_2_ nanoparticles [19,20], into silicone-containing polyurethane resins, resulting in rough surfaces with micro- and nanostructures like those on the surface of lotus leaves [21], which makes these coatings superhydrophobic. Zhou et al. [22] obtained a superhydrophobic coating with a contact angle of 163° and a roll angle of 1.4° by using a silane coupling agent to modify rough SiO_2_ and covering it on a substrate by dip-coating method, which was in turn coated with stearic acid. Wooden products are used in a wide range of applications. In order to protect wood products exposed to the outdoors, reduce their exposure to the external environment and extend their service life, applying superhydrophobic coatings to their surfaces is a suitable method [23,24]. However, due to the presence of hydrophobic PDMS in the waterborne polyurethane system, superhydrophobic coatings prepared by physical blending of PDMS and SiO_2_ particles with systems would generally make the emulsion susceptible to phase separation, resulting in poor stability.

Currently, the synthesis of waterborne polyurethane coatings is overly dependent on petroleum-based energy, and this dependence leads to the excessive consumption of non-renewable energy sources, which in turn leads to serious environmental problems. Therefore, the synthesis of waterborne polyurethane coatings using biomass-based polyols instead of petroleum-based polyols has become an environmentally friendly route [25,26,27]. Among many biomass-based polyols, castor oil, as the only natural vegetable oil with polyhydroxyl groups [28], has become an ideal material for the synthesis of polyurethanes instead of conventional petroleum-based polyols reacted with diisocyanates.

In this study, inspired by the hydrophobic effect of hydroxyl-terminated polydimethylsiloxane (OH−PDMS) and micro-nanoscale heptadecafluorodecyltrimethoxysilane-modified SiO_2_ particles (FAS−SiO_2_) [10,15,22], these were introduced simultaneously into the castor-oil-based waterborne polyurethane emulsion to elicit superhydrophobicity through the synergistic effect. This waterborne polyurethane system was originally derived from biomass and was stable without phase separation. The superhydrophobic coating surface with dense micro- and nanostructures was expected to reduce the contact area of water droplets on the coating and prevent water droplets from completely infiltrating the surface, thus possessing self-cleaning characteristics. This innovative approach offers guidance for the development of novel coatings with self-cleaning functionality.

## 2. Materials and Method

### 2.1. Materials

Isophorone diisocyanate (IPDI, analytical grade, McLean, Shanghai, China), castor oil (C.O., analytical grade, hydroxyl value of 164 mg KOH/g, McLean, Shanghai, China), MDEA (analytical grade, McLean, Shanghai, China), acetone (analytical grade, Tianjin Tianli Chemical Reagent Co., Ltd., Tianjin, China), hydroxyl-capped polydimethylsiloxane (OH−PDMS, analytical grade, Aladdin, Shanghai, China), dibutyltin dilaurate (DBTDL, analytical grade, Maclean, Shanghai, China), pentaerythritol triacrylate (PETA, analytical grade, Maclean, Shanghai, China), glacial acetic acid (analytical grade, Maclean, Shanghai, China), deionized water (prepared in the laboratory), fumed nanoparticles of silicon dioxide (with particle sizes of about 10 nm, 100 nm, and 1 μm, McLean, Shanghai, China), heptadecafluorodecyltrimethoxysilane (FAS−17, Maclean, Shanghai, China), and the photoinitiator IRGACURE1173 (analytical grade, Wuxi A, B, C, D Biotechnology Co., Wuxi, China) were used in this study.

Acetone (AC) was dried using an activated 4A molecular sieve for 24 h before use, and C.O., OH−PDMS, and MDEA were dried under vacuum at 100 °C for 2 h. Acetone is used as the main solvent for synthesizing polyurethanes.

### 2.2. Synthesis of Silicone-Modified Castor-Oil-Based UV-Curable Waterborne Polyurethane (SCWPU)

**Figure 1:**
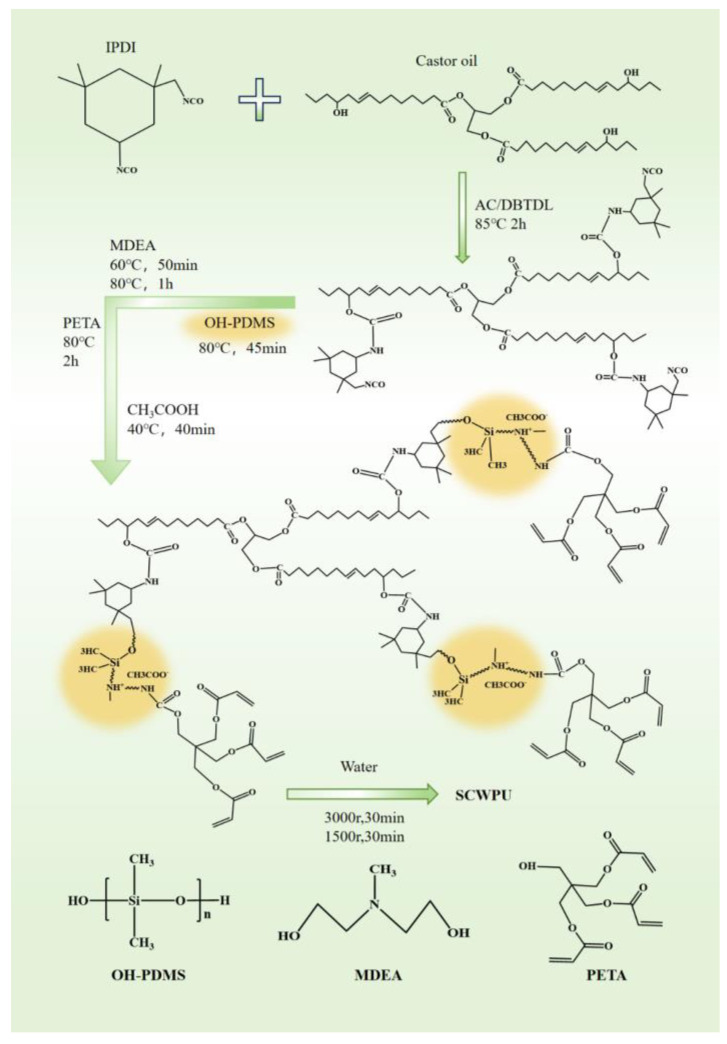
Reaction for the preparation of silicone-modified castor-oil-based UV-curable waterborne polyurethanes (SCWPUs).

First, 51.225 g of IPDI was added to a 500 mL four-necked flask with nitrogen gas, and the flask was fitted with a spherical condenser tube, stirring paddle, and constant-pressure dropping funnel. Then, 20.715 g of C.O. was added dropwise through the funnel, and the polyurethane prepolymer solution was synthesized via reaction for 2 h at 85 °C in a constant-temperature oil bath. Next, 11 g of OH−PDMS was added dropwise, and after 30 min of reaction, the thermostatic oil bath was cooled down to 65 °C; then, 8 g of MDEA was added dropwise after 50 min of reaction, the oil bath temperature was raised to 80 °C; then, the reaction was allowed to proceed for 1 h. Next, 5 g of PETA was added dropwise as a capping agent, and the reaction was carried out for 2 h. When the proportion of the –NCO group reached the theoretical value (titrated by di-n-butylamine), the temperature was lowered to 40 °C and 4.03 g glacial acetic acid was added for 40 min to neutralize the reaction until the system was neutral. An appropriate amount of acetone was continuously added to adjust the viscosity during the reaction. Finally, distilled water was added under high-speed shear force for emulsification, and then the acetone solvent was removed by distillation under reduced pressure to prepare the SCWPU emulsion containing OH−PDMS chain segments with a solid content of about 30%. In the system, the molar ratio of n(−OH) to n(−NCO) was 0.99. In total, five groups of SCWPU emulsions were prepared, and OH−PDMS was added to the polyurethane with mass fractions of 7%, 8%, 9%, 10%, and 11%; the corresponding polyurethanes were denoted as SCWPU-7, SCWPU-8, SCWPU-9, SCWPU-10, and SCWPU-11, respectively. The reactions are presented in Figure 1.

### 2.3. Preparation of FAS−17—Modified Silica Nanoparticles

First, an aqueous ethanol solution with a volume ratio of water to ethanol of 1:9 was prepared, and 100 mL of the solution and 4.5 g of FAS−17 were added into a beaker. The contents of the beaker were stirred well using a magnetic stirrer, and acetic acid was added to adjust the pH value of the solution to between 3 and 5, followed by spontaneous hydrolysis for 1 h at 25 °C to prepare liquid A. Subsequently, 5.03 g of SiO_2_ nanoparticles with particle sizes of 10 nm, 100 nm, and 1 μm were separately added to 500 mL of an aqueous ethanol solution with a volume ratio of water to ethanol of 1:9 and dispersed through ultrasonic dispersion for 30 min to obtain liquid B. Liquid A was mixed with liquid B and added to a three-necked flask with a magnetic stirring and stirred at 50 °C for 6 h. The product was subjected to rotary evaporation and placed in a vacuum drying oven at 40 °C for 10 h for drying. Fluorine-modified hydrophobic silica particles were obtained by milling, and the corresponding particles were denoted FAS−SiO_2_—10 nm, FAS−SiO_2_—100 nm, and FAS−SiO_2_—1μm, respectively. These modified nanoparticles were then added as fillers to the SCWPU-11 resin matrix at a particle size ratio of 1:1:1.

### 2.4. Preparation of Films and Coatings

Different mass ratios of FAS−SiO_2_ were ultrasonically dispersed in ethanol for 15 min and added to measured volumes of SCWPU, with rapid stirring with a magnetic stirrer for 2 h; the liquid was then dispersed by ultrasonic waves for 20 min to obtain a coating with a solid content of about 15%. Then, 3 wt% of photoinitiator 1173 was added to the coating solution and stirred with a magnetic stirrer for 2 h. Finally, the stirred coating was bottled and stored away from the light.

Film preparation: The configured coatings were cast in 45 mm × 12 mm × 1 mm polytetrafluoroethylene molds and round silicone molds with a diameter of 6 cm. The films were kept at room temperature for 1 day and then dried in an infrared oven at 50 °C for 5 h to ensure that water was completely removed. Finally, they were cured under UV−LED light at a wavelength of 395 nm. The films were kept at 25 °C for 7 days to dry, and then a range of properties of coated films were tested.

Wood coating preparation: The ashlar veneer (250 mm × 96 mm × 15 mm) was sanded with 320-mesh sandpaper in the direction of the wood grain, and the board was dried in an oven at 50 °C for 1 day. After the pretreatment of the boards, the configured coating was uniformly applied to the surface of the wood flooring samples and pre-dried in an oven at 50 °C for 1 min. The boards with coatings were then removed and cured under a UV−LED light source of 15,000 mJ/cm^2^ for 5 s (optical energy density of 400 mW/cm^2^, irradiation distance of 10 cm). The above procedure was repeated three times. The final thickness of the film was about 70–80 μm. The film was stored at room temperature for 10 days, and then the performance of the wood coating was analyzed.

### 2.5. Tests and Characterization

The samples were analyzed using a Nicolet iS50 Fourier Transform Infrared Spectrometer (FTIR) at room temperature, and the test method was either the thin-film method or the KBr compact method, depending on the current state of the sample, with the wavelengths ranging from 400 to 4000 cm^−1^ and a resolution of 2 cm^−1^.

A Zetapals 0–555 laser particle size analyzer was used to test the particle size distribution of the waterborne polyurethane (WPU) emulsion. The storage stability of the emulsion was determined with reference to CB/T 6753.3-1986 “Test Method for Storage Stability of Paint”. A small amount of emulsion was placed in a 5 mL centrifuge tube, and the rotational speed was set at 3000 r/min for 15 min; then, the presence of a precipitate was identified. The microscopic morphology of the SCWPU emulsion was observed by using a JEM2100 transmission electron microscope, and the morphology of latex particles was observed by TEM.

The QUANTA200 SEM was used to observe the morphology of the SCWPU emulsion and SCWPU film surfaces, and energy-dispersive spectroscopy (EDS) was performed to observe the elemental composition of the composite films. The degree of microphase separation, roughness, and morphology of the surface of the adhesive film were observed using AFM (5 μm × 5 μm) at 25 °C, and different areas of the sample were scanned using the knockdown mode at a scanning frequency of 1 Hz. The data were analyzed using the Nano Scope Analysis software (v180r1). The water contact angle (WCA) values were measured using a contact angle analyzer model OCA20 at 25 °C: 4 μL of water droplets were placed on the surface of the SCWPU-coated membrane. The WCA values were recorded by the analysis software, and the state diagram of the water droplets on the membrane surface after stabilization was retained. The hysteresis angle (HA) was measured by the addition and subtraction method. Three measurements were taken at three different points for each sample, and the average values were recorded.

The tensile properties were analyzed using an electronic universal testing machine. Three parallel tests were performed for each set of samples. The dynamic mechanical analysis (DMA) of the film was carried out using a DMA Q800 dynamic mechanical thermal analyzer in the tensile mode, and the temperature range was from −50 to 100 °C with a heating rate of 5 °C/min, a tensile frequency of 1 Hz, and an oscillation displacement of 20 μm. Further, thermogravimetric analysis (TG) was carried out using the TG209F3 Nevio thermogravimetric analyzer to test the heat resistance of the sample film, using argon gas as a protective gas, and the heating rate was 10 °C/min with a test temperature of 25–600 °C.

The film properties on the UV-cured wood paint film were tested after 7 days without disturbance. The hardness of the paint film was determined according to GB/T 6739-2006, and the adhesion grade of the wood paint film was tested according to GB/T 4893.4-2013. The gloss of the paint film was tested according to GB/T 4893.6-2013, and the roughness of the paint film was measured using the Surtronic Duo Surface Roughness Tester. The above testing method was conducted three times for each sample, and the average value of the three replicates was then calculated. The thickness of the wood coating is tested according to GB/T 13452.2-2008, and each sample is tested three times to take the average value.

## 3. Results and Discussion

### 3.1. Structural Characterization and Elemental Composition of OH−PDMS—Modified Castor-Oil-Based Waterborne Polyurethane

**Figure 2:**
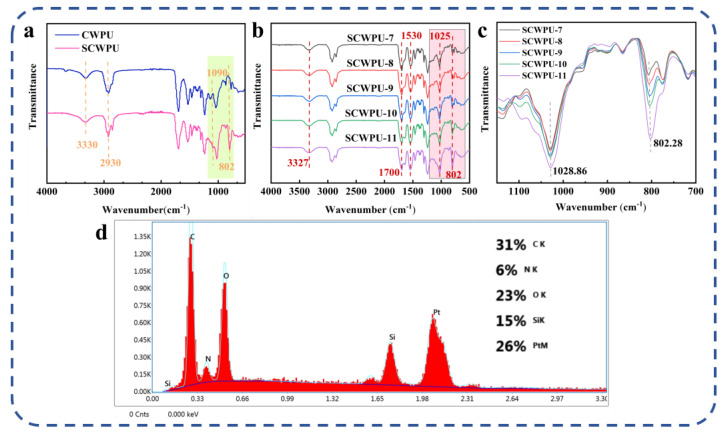
Structural and elemental characterization of SCWPU: (**a**) FTIR results for polyurethane film before and after the addition of OH−PDMS. (**b**) Comparison of FTIR spectra of SCWPU specimens with different OH−PDMS contents. (**c**) FTIR spectra (700–1150 cm^−1^) of SCWPU specimens with different OH−PDMS contents. (**d**) SCWPU surface composition.

The structure and elemental composition of SCWPU were characterized using FTIR and EDS analyses (Figure 2). As shown in Figure 2a,b, the peak at 3330 cm^−1^ is the stretching vibration peak of N–H in the urethane moiety, and the peak at 2930 cm^−1^ is the stretching vibration absorption peak of C–H in the methyl group; further, the absorption peak at 1530 cm^−1^ is related to the stretching vibration peak of –NH in the urethane moiety, and the characteristic absorption peak of C=O appears at 1700 cm^−1^. These results indicate the successful synthesis of waterborne polyurethane [29]. The absence of the characteristic absorption peak of the –NCO group at 2270 cm^−1^ indicates that –NCO in the reaction system has been completely consumed [30]. In contrast to castor-oil-based waterborne polyurethane (CWPU), the SCWPU shows two characteristic silicone peaks at 1090 cm^−1^ and 802 cm^−1^ corresponding to the asymmetric and symmetric telescopic vibration peaks of Si–O–Si and Si–O–C, respectively, and the characteristic absorption peak of Si–CH_3_ appears at 1025 cm^−1^, indicating that a silicone-containing chain segment had been successfully introduced into the system and thus confirming the synthesis of SCWPU.

As shown in Figure 2b,c, the peak areas of organosilica at 802 cm^−1^ and 1025 cm^−1^ increased gradually with the increase in the OH−PDMS content in the system, and the peak areas of SCWPU-11 at 1028 cm^−1^ and 802 cm^−1^ were the largest, which was consistent with the mechanisms governing the system.

As per the EDS spectra (Figure 2d), in addition to the elements contained in the polyurethane material, silicon is present on the surface of the material with the proportion reaching 15%. This indicates the successful incorporation and uniform dispersion of OH−PDMS within the chain segments of polyurethane molecules. The enrichment of silicon on the surface of SCWPU films suggests the same for the hydrophobic group (methyl group) in OH−PDMS, which contributes to the reduction in its surface energy, resulting in desirable surface properties [31].

### 3.2. Stability of OH−PDMS—Modified Castor-Oil-Based Waterborne Polyurethane Emulsions

**Figure 3:**
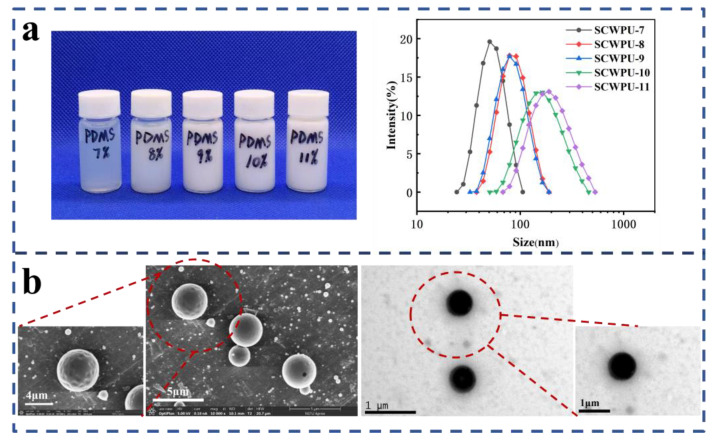
Stability and bacterial inhibition of emulsions: (**a**) appearance and particle size of SCWPU emulsions with different OH−PDMS contents; (**b**) SEM (10,000× and 20,000× magnification) and TEM (10,000× magnification) images of SCWPU emulsions.

The emulsion particle size, particle morphology, storage stability of SCWPU emulsions with different OH−PDMS contents were analyzed, and the results are shown in Figure 3. The macroscopic emulsion properties of SCWPU are listed in Table 1.

Figure 3a shows the particle size and macroscopic properties of SCWPU specimens with different OH−PDMS contents. The particle size of SCWPU emulsions gradually increased with the OH−PDMS content: when the OH−PDMS content increased from 7% to 11%, the particle size of the emulsion increased from 51.24 nm to 186.27 nm, the transparency of the emulsion was reduced from slightly yellow to milky white, the phenomenon of bluish light reflectance was weakened [32], the particle-size polydispersity index (PDI) increased, and there was no double peak phenomenon. These results indicate that SCWPU was uniformly dispersed in the water and had a narrow particle size distribution with good stability. As can be seen from Table 1, all emulsions showed excellent storage stability (more than six months), and the solid content was between 27% and 30%.

Figure 3b shows the SEM and TEM characterization results for SCWPU emulsions. The microscopic morphology of the latex particles can be clearly seen, and all the latex particles exhibit a regular spherical shape with distinct boundaries [33]. Further, the particles exhibit a uniform size (1 µm) and distribution.

### 3.3. Preferred Formulations for SCWPU Coating Film with Hydrophobic Properties

In this study, SCWPU resin films with different OH−PDMS contents were characterized and their WCAs, surface morphology, tensile properties, thermodynamic properties (DMA), thermal weight loss properties (TG), and varnish properties were assessed.

#### 3.3.1. Water Contact Angle and Surface Morphology of SCWPU Coating

**Figure 4:**
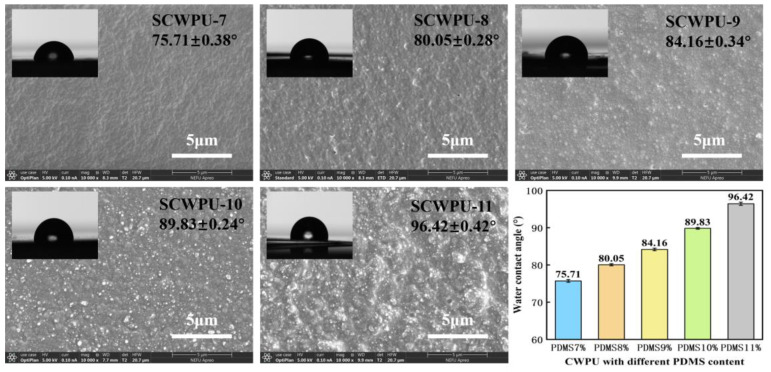
Contact angle and surface morphology of SCWPU with different OH−PDMS contents (SEM images at 10,000× magnification).

Figure 4 shows the WCAs of SCWPU specimens with different OH−PDMS contents and the surface morphology observed by SEM at 5 μm scale. The WCAs of the SCWPU films gradually increased from the initial (75.71 ± 0.38)° to (96.42 ± 0.42)° with the increase in the OH−PDMS content, and the wettability of the coating surface changed, transforming the surface from hydrophilic to hydrophobic. This is because the introduction of low-surface-energy organosilicon compounds on the surface of the polyurethane coating is conducive to the reduction in the surface energy [16]. The SEM results show that the surface roughness of the SCWPU film gradually increased with the OH−PDMS content, changing from a slightly rough texture to the appearance of agglomerated latex particles. The WCA no longer increases when the silicon content on the coating surface approaches saturation.

#### 3.3.2. Mechanical and Thermodynamic Properties of SCWPU-Coated Film

**Figure 5:**
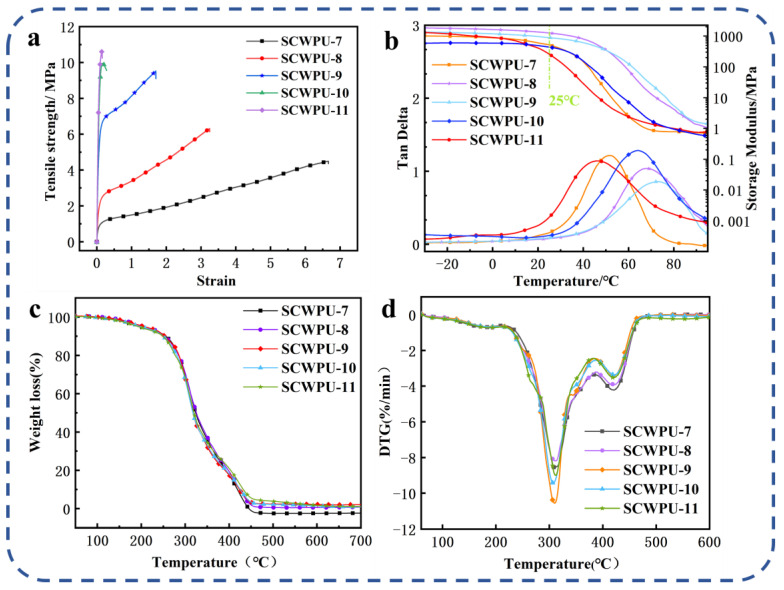
Mechanical and thermomechanical properties of SCWPU: (**a**) stress–strain curve, (**b**) DMA results, (**c**) TG results, and (**d**) DTG results.

From Figure 5a and Table 2, the tensile strength and Young’s modulus of SCWPU films can be seen to gradually increase with the OH−PDMS content, while the elongation at break gradually decreases. SCWPU-7 has the lowest tensile strength of 4.49 MPa, and the highest elongation at break of 665.79%. The tensile strength of SCWPU-11 and the Young’s modulus are as high as 10.65 MPa and 167.92 MPa, respectively, while the elongation at break is only 15.26%. As the OH−PDMS content in the polyurethane system gradually increases, the increase in the number of Si–O bonds leads to an increase in the cross-linking density of the system, and the formation of intermolecular hydrogen bonds impedes the relative sliding of the molecules, thus improving the tensile strength and increasing the modulus of elasticity [34]. However, since the added OH−PDMS partially replaces castor oil, the content of soft segments (i.e., castor oil) in the system decreased, and the flexibility of the film was reduced; furthermore, the rigidity of the hard segments (i.e., the carbamate groups) of the SCWPU molecular chain gradually increased with the OH−PDMS content, so the elongation at break decreased. Thus, the addition of OH−PDMS can improve the mechanical properties of polyurethane materials.

In Figure 5b, the DMA results for SCWPU [the energy storage modulus (E) and loss factor (tan θ)] are shown, and further details are listed in Table 3, where Tg represents the glass transition temperature. The crosslinking density (υe) was calculated based on the rubber elasticity:

(1) $$\upsilon e=E^{\prime }/3RT$$

where E′ is the storage modulus at 20 °C above Tg, R is the gas constant, and T is the absolute temperature [33,35,36]. Since the material will be used at room temperature (25 °C), most of the time, the energy storage modulus of the material at 25 °C is very important [33]. As the OH−PDMS content was increased from 7% to 11% at 25 °C, the energy storage modulus of the films showed a trend of increasing from 515.96 MPa to 1259.21 MPa and then decreasing to 258.57 MPa. The increase in the energy storage modulus was attributed to the C=C double bonds and Si–OH groups. The Si–OH groups in siloxanes condense to form an interpenetrating network with the polymer phase, increasing the crosslinking density of the system [34]. Further, the C=C double bond acts as an active site for resin crosslinking during UV curing, thus increasing the crosslinking density. Although a higher crosslinking density limits the movement of SCWPU chains and leads to higher E values, when the content of the soft segment C.O. is decreased with increasing OH−PDMS content, the flexibility of the material, and the energy storage modulus decrease. Hence, the above results are mainly attributed to the combined effects of the C.O. and OH−PDMS contents.

The degree of glass transition of SCWPU resins is influenced by the crosslinking density and the number of suspended chains [37]. The Tg is high because of the condensation of the Si–OH groups in the siloxanes to form an interpenetrating network with the polymer phase, resulting in an increase in the crosslinking density of the system. Although a higher crosslinking density will restrict the movement of SCWPU chains and lead to a higher Tg, the increase in the number of suspended chains on SCWPU with the increase in OH−PDMS will lead to a decrease in Tg since the suspended chains will act as plasticizers, reduce the rigidity of the material, and increase the resin fluidity. The SCWPU-11 film has the highest number of suspension chains, resulting in a decrease in Tg. Therefore, the above results can be mainly attributed to two opposite factors: crosslinking density and suspension chain.

The TGA curves and DTG curves of SCWPU films are shown in Figure 5c,d, and the TGA data are listed in Table 3. All the sample films have a similar thermal decomposition process, and the thermal degradation process can be divided into three stages: The first stage at 150–250 °C is the breakage of some residual small molecules and fatty acid chains in the system. In the second stage, at 250–450 °C, the fastest rate of degradation is observed, which mainly involves the decomposition of the film’s hard section, and the unstable ethyl carbamate bond dissociates in this stage. Finally, in the third stage above 450 °C, SCWPU films undergo further thermal oxidation in air [35]. As the OH−PDMS content was increased from 7% to 11%, the initial decomposition temperatures of the materials were around 200 °C, with good thermal stability and a certain upward trend, which satisfies the temperature conditions for application in natural outdoor conditions, and the amount of residue also increased from 0.002% to 6.44%. This indicates that the addition of OH−PDMS can effectively prevent the decomposition of polyurethane, which is attributed to the condensation of Si–OH groups in the structure of PDMS, resulting in the formation of an interpenetrating network with the polymer phase and an improvement in the cross-linking density. Further, the energy of the inter-bonding of Si–O–Si is higher than that of C–O–C, because Si–O–Si absorbs part of the heat during the temperature rise, thus improving the thermal stability of the polyurethane material. There was no significant difference in the maximum degradation temperature (Tdmax) of SCWPU films, which were all around 310 °C.

#### 3.3.3. Performance of SCWPU Coatings

The film properties of the SCWPU coatings are shown in Table 4. The hardness of all the SCWPU coatings analyzed by performing the pencil hardness test was 4H. With the increase in OH−PDMS content, the dispersion of the latex particles in the coatings decreased due to the increase in the average particle size of the system, which led to an increase in the roughness of the system and a decrease in the gloss. The adhesion of all the coatings was grade 0, which may be due to the hydroxyl groups in the polyurethane system reacting with the hydroxyl groups on the surface of the wood to form hydrogen bonds [38], which provide a good bonding strength and show high adhesion; as wood is composed of many cells with tubular pores [36], low-viscosity coatings exhibit good mobility and penetration into the wood surface, and the coatings can form a large number of anchor points, further improving the adhesion.

In summary, based on the various paint film properties, along with the hydrophobicity, mechanical properties, and thermal stability of the material, SCWPU-11 was identified as the optimal base resin to continue with the construction of the superhydrophobic coating.

### 3.4. Characterization and Analysis of Superhydrophobic Coatings

Based on the optimal formula of SCWPU-11 as determined in the above experiment, three different particle sizes (approximately 10 nm, 100 nm, and 1 μm) were used in this experiment. The effect of FAS−SiO_2_ addition on the hydrophobicity of the coating was investigated by changing the amount of FAS−SiO_2_ added. Based on the quality of the resin, the particle addition amounts were 10%, 20%, 30%, 40%, and 50%. The coatings were named SCWPU/FAS−SiO_2_—10%, SCWPU/FAS−SiO_2_—20%, SCWPU/FAS−SiO_2_—30%, SCWPU/FAS−SiO_2_—40%, and SCWPU/FAS−SiO_2_—50%.

#### 3.4.1. FTIR and EDS Analysis

**Figure 6:**
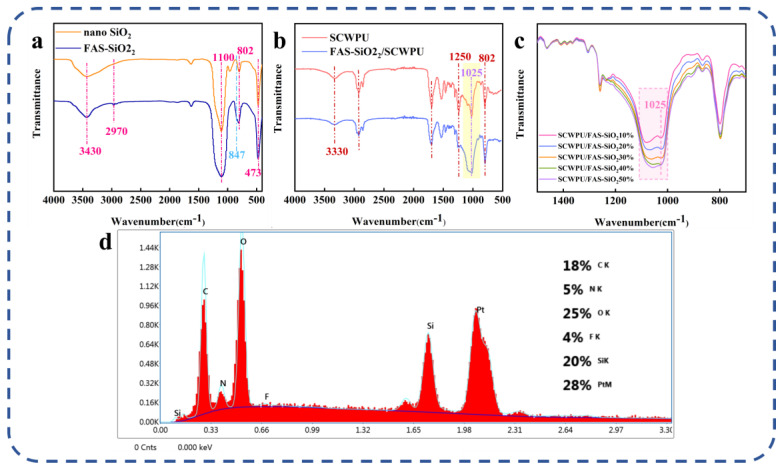
Structural and elemental characterization: (**a**) FTIR results for SiO_2_ nanoparticles before and after modification. (**b**) FTIR results before and after addition of FAS−SiO_2_ particles. (**c**) FTIR results for SCWPUs with different FAS−SiO_2_ contents. (**d**) EDS results for coating surface.

As can be seen from Figure 6a, the telescopic vibration peak of –OH appeared at 3430 cm^−1^, and the telescopic vibration absorption peak of –CH– appeared at 2970 cm^−1^. Furthermore, there are anti-symmetric telescopic vibration peaks, symmetric telescopic vibration peaks, and a characteristic absorption peaks at 1100 cm^−1^, 802 cm^−1^, and 473 cm^−1^, respectively. These correspond to the bending vibration absorption peaks of Si−O−Si and Si−O−C. More importantly, compared to the unmodified nano-SiO_2_ curves, an additional absorption peak of the deformed vibration absorption peak of −CF− was observed at 847 cm^−1^ in the FAS−SiO_2_ curves; this demonstrates that the modification of the nano SiO_2_ particles is successful [39].

Figure 6b shows that the N–H stretching vibration peak at 3330 cm^−1^ and the C–H stretching vibration absorption peak at 2930 cm^−1^ are characteristic peaks of polyurethane, which proves that both polymers have the molecular structure of polyurethane [40]. Compared to the SCWPU curve, the FAS−SiO_2_/SCWPU curve exhibits a more pronounced characteristic absorption peak of Si−O−Si at 1025 cm^−1^ in the yellow region. Additionally, the characteristic absorption peaks and bending vibration peaks of Si−CH_3_ at 1250 cm^−1^ and 802 cm^−1^ are observed. Stronger characteristic bands appear in the 1025–1250 cm^−1^ region and are attributed to the characteristic absorption peaks of C–O–C with C–F and –CF_2_ groups. The FTIR plots corresponding to different FAS−SiO_2_ contents (Figure 6c) show that, as the FAS−SiO_2_ content increases, the stretching vibration peak and symmetric stretching vibration peak associated with Si–O–Si and Si–O–C and characteristic absorption peaks’ height corresponding to C–O–C, C–F, and –CF_2_ groups (pink area) gradually increase; this is consistent with the system’s pattern of change, indicating the successful introduction of FAS−SiO_2_ into the SCWPU system [40]. In the EDS spectra (Figure 6d), the proportion of fluorine on the surface of the material reaches 4%, and the proportion of silicon on the surface of the material reaches 20%, which indicates that FAS−SiO_2_ has been successfully introduced and homogeneously dispersed in the polyurethane molecular chain segments.

#### 3.4.2. Water Contact Angle and Microstructure Analyses

**Figure 7:**
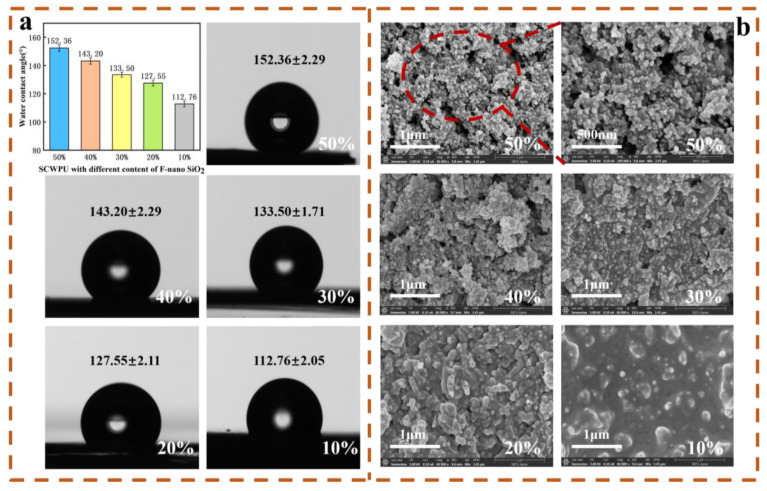
Wettability and surface morphology of SCWPU/FAS−SiO_2_ coating: (**a**) water contact angle and (**b**) SEM images (SEM magnification is 60,000× for 10–50% FAS−SiO_2_, and enlarged image magnification of 100,000×).

As can be seen from Figure 7a,b, with the addition of 10–50% FAS−SiO_2_ particles, the WCA of the coatings gradually increased from (112.76 ± 2.05)° to (152.36 ± 2.29)°, resulting in the transition from a hydrophobic coating to a superhydrophobic one [41]. The SEM images show that the surface roughness of the coating gradually increases with the FAS−SiO_2_ content. With 10% FAS−SiO_2_ particle addition, the surface of the coating shows widely dispersed microstructures, but because the added amount is small, most of the FAS−SiO_2_ particles are still encapsulated in the inner part of SCWPU and not exposed on the surface. With the continuous increase in the added particle amount, more particles are enriched on the surface of the polyurethane, but due to the agglomeration of some of the particles, the coating exhibits only micrometer-level roughness structures. However, when the amount of FAS−SiO_2_ particles is 40%, the WCA of the coating reaches (143.2 ± 2.29)° only, which does not correspond to the superhydrophobic state. As the amounts of particles added is further increased to 50%, the coating surface with the accumulated particles becomes similar to the surface of the lotus leaf “papillae” micro- and nanostructures. As can be seen at a 500-nm SEM magnification, the surface of the coating has dense spherical particles that are not encapsulated in the coating substrate. At this point, a more regular micro-nanostructure formed on the coating surface with a contact angle of (152.36 ± 2.29)° and a roll-off angle of (4.9 ± 1.0)°. This gave the coating the ability to produce a “lotus leaf effect” and exhibit superhydrophobic properties [21].

#### 3.4.3. Two- and Three-Dimensional Characterization of AFM

**Figure 8:**
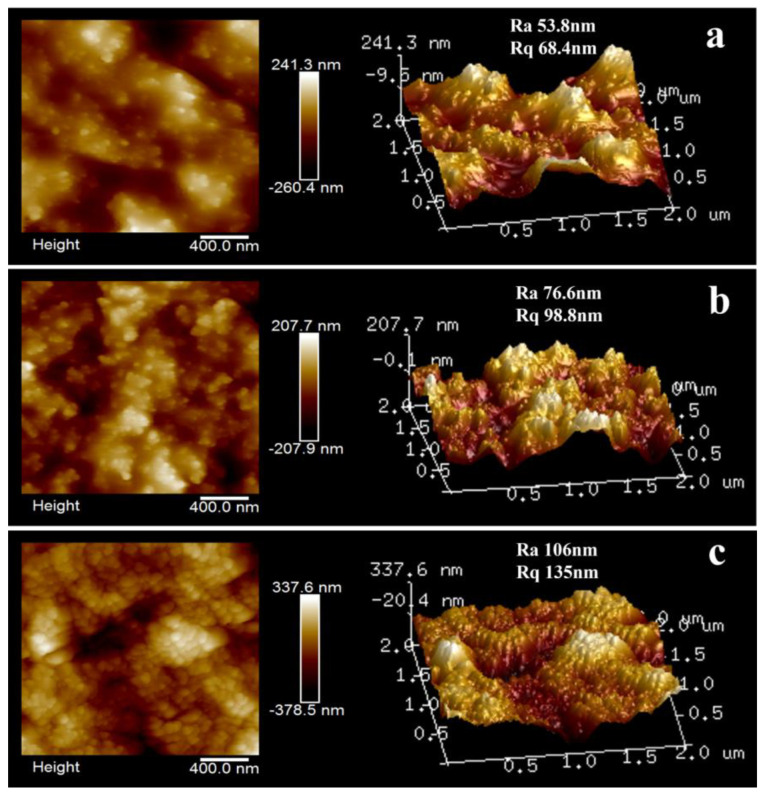
Microscopic morphology of SCWPU/FAS−SiO_2_ coatings: 2D and 3D height maps of the surface morphology of the coatings with 30% (**a**), 40% (**b**), and 50% (**c**) FAS−SiO_2_ particles.

The surface morphology of several typical SCWPU/FAS−SiO_2_ coatings was observed using AFM. As shown in Figure 8a, the morphology of the surface of the coatings with different particle contents obviously differ. When the FAS−SiO_2_ content is 30%, the surface of the coatings exhibits bright adhering protrusions. These may be microscale spherical particles formed from different nanoscale FAS−SiO_2_ particles migrating to the surface of the coatings and being encapsulated in the SCWPU resin, as a result of which the bumps appear to be larger and unevenly dispersed, and the structures resulting in the rough surface are not dense. When the content of FAS−SiO_2_ particles is increased to 40%, the contrast between peaks and valleys on the surface of the coating becomes increasingly significant, the surface roughness increases, and the bumps become smaller in size and more evenly distributed on the surface of the coating. When the content of FAS−SiO_2_ particles is increased to 50%, clear micro- and nanostructures can be seen on the surface of the coating, and the raised particles are uniformly distributed and densely arranged, forming a micro- and nanoscale papillate structure similar to that on the surface of the lotus leaf. This is caused by the migration of fluorine-containing SiO_2_ particles and the synergistic effect of different particle sizes. When the FAS−SiO_2_ content is low, the Rq value of the coating is small; meanwhile, when the FAS−SiO_2_ content is increased, the Rq value increases significantly from 68.4 nm to 135 nm. Therefore, when the FAS−SiO_2_ content reaches 50%, the surface roughness of the coating is adequate to result in superhydrophobic structural characteristics.

#### 3.4.4. Characterization of Coating Self-Cleaning Properties

**Figure 9:**
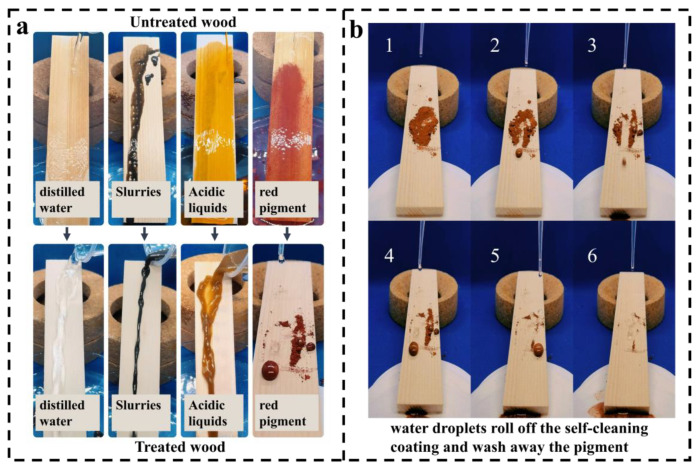
Characterization of the self-cleaning properties of coatings: (**a**) Sliding of different liquids on the surface of coated and uncoated wood. (**b**) Self-cleaning action of SCWPU/FAS−SiO_2_ coating (Numbers 1–6 indicate the sequence of the process of water droplets falling on the surface of the coating).

As shown in Figure 9a, the self-cleaning performance of the coatings was examined by comparing the observed sliding traces of different liquids on the surfaces of coated and uncoated wood samples [42]. Further, in Figure 9b, the self-cleaning function of the coatings was verified by uniformly spreading a brick-red color pigment on the surface of the wood and tilting the wood samples at an angle of 45°. A dropper was used to apply water dropwise onto the surface of the wood, and the water droplets were allowed to roll down continuously from the top of the wood coating [41]. This setup enables one to observe how the water droplets removed the pigment particles from the coating surface using its self-cleaning ability.

Figure 9a shows that the SCWPU/FAS−SiO_2_ coating has self-cleaning properties when exposed to different liquids such as water, mud, and acidic liquid. Figure 9b shows that the water droplets rolled down along the surface of the coating and removed the pigment particles on the surface of the coating while rolling down; thus, the coating surface became clean after a certain number of water droplets rolled down. In addition, the original superhydrophobicity of the coating remains unchanged after 10 cycles of self-cleaning tests. These test results show that the coating exhibits excellent self-cleaning performance.

#### 3.4.5. Characterization and Analysis of Coating Film Properties

Table 5 lists the paint film properties of coatings with different FAS−SiO_2_ contents. As the FAS−SiO_2_ content increases, the gloss of the paint film decreases significantly, the roughness increases, the adhesion decreases, and the hardness increases. The decrease in gloss is attributed to the increase in roughness: an increasing number of micro- and nanostructures appeared on the surface of the coating due to the enrichment and stacking of FAS−SiO_2_ particles on the coating. The hardness, as determined via the pencil hardness test, increased from 4H to 5H. This was due to the stacking of FAS−SiO_2_ particles improving the strength of the coating surface. Furthermore, the adhesion of the paint film decreased from 0 to 2.

## 4. Conclusions

In this study, hydroxyl-terminated polydimethylsiloxane (OH−PDMS) was introduced into castor-oil-based waterborne polyurethanes, and a siloxane-modified castor-oil-based UV-curable waterborne polyurethane (SCWPU) system was designed. The coating with optimal hydrophobicity, namely, SCWPU-11 was selected, and different amounts of micro- and nanoscale FAS−SiO_2_ particles were introduced into this system to prepare the superhydrophobic coating. The following characterization and analysis results were obtained.

All the emulsions exhibited good stability, with a preservation period of more than 6 months. As the OH−PDMS content was increased from 7 wt% to 11 wt%, the average particle size of the emulsions increased from 51.24 nm to 186.27 nm. When the OH−PDMS content reached 11%, the contact angle of the SCWPU films increased to (96.42 ± 0.42)°, indicating improved hydrophobicity. The films also showed good thermal and mechanical properties, with a high tensile strength of 10.65 MPa. In addition, all coatings exhibited excellent adhesion, and the hardness reached 4H. In the case of FAS−SiO_2_ particles, when three sizes of particles (100 nm, 1 μm, and 10 nm) at a ratio of 1:1:1 were added to SCWPU-11 with a total additive amount of 50 wt%, the water contact angle of the coating reached (152.36 ± 2.29)° and the roll-off angle reached (4.9 ± 1.0)°. This demonstrates a significant increase in hydrophobicity, indicating improved water repellency. The coating surface exhibited roughness with micro- and nanostructures, resulting in superhydrophobicity. In this study, a stable SCWPU emulsion was synthesized by substituting petroleum-based polyols. Subsequently, a biomass-derived UV superhydrophobic coating with self-cleaning characteristics was prepared. Compared to other similar coatings, SCWPU is not only environmentally friendly but also has the potential to resist moisture, water, and stains when applied to wood products exposed to outdoor environments.

## Figures and Tables

**Table 1:** Macroscopic properties of SCWPU emulsions with different OH−PDMS contents.

Emulsion Properties	SCWPU-7	SCWPU-8	SCWPU-9	SCWPU-10	SCWPU-11
Stability	unstratified	unstratified	unstratified	unstratified	unstratified
Color	light yellow	milky white	milky white	milky white	milky white
Bluish light phenomenon	++	++	+	+	+
Solid content/%	29.32	28.64	27.81	28.93	27.15
Storage Stability	>6 months	>6 months	>6 months	>6 months	>6 months
Transparency	transparent	semitransparent	semitransparent	non-transparent	non-transparent
Average particle size/nm	51.24	82.42	76.66	154.50	186.27
Polydispersity index	0.069	0.078	0.109	0.175	0.191

Note: “+” indicates a weak bluish light reflectance phenomenon; “++” indicates a strong bluish light reflectance phenomenon.

**Table 2:** Tensile strength, Young’s modulus and elongation at break of SCWPU specimens.

Samples	Tensile Strength/MPa	Elongation at Break/%	Modulus of Elasticity/MPa
SCWPU-7	4.49 ± 0.25	665.78 ± 28.87	0.55 ± 0.02
SCWPU-8	6.31 ± 0.41	324.66 ± 16.80	11.94 ± 0.09
SCWPU-9	9.53 ± 0.35	169.70 ± 12.37	82.57 ± 1.36
SCWPU-10	9.95 ± 0.44	17.54 ± 0.21	147.99 ± 2.06
SCWPU-11	10.65 ± 0.61	15.26 ± 0.16	167.92 ± 2.93

**Table 3:** DMA and TGA data for SCWPUs.

Samples	TGA					DMA			
	T_d5%_	T_d10%_	T_d50%_	T_dmax_	Char Yield	E	Tg	E’	υe
	(%)	(%)	(%)	(%)	(%)	(at 25 °C, MPa)	(°C)	(at Tg + 20 °C, MPa)	(mol/m^3^)
SCWPU-7	197.86	255.62	323.97	309.14	0.002	515.96	51.74	0.85	99.16
SCWPU-8	205.67	253.41	325.43	311.92	2.849	1259.21	67.83	1.66	183.91
SCWPU-9	206.97	253.22	317.80	309.84	3.964	886.42	73.09	1.47	160.49
SCWPU-10	198.50	249.60	317.60	307.38	4.218	436.45	64.13	0.80	89.44
SCWPU-11	193.10	249.05	320.20	311.95	6.440	258.57	46.33	1.63	192.50

**Table 4:** Properties of SCWPU coatings.

	Glossiness (60 °C)	Adhesion	Pencil Hardness	Roughness (µm) Ra
SCWPU-7	81.67	0	4H	0.20
SCWPU-8	68.30	0	4H	0.21
SCWPU-9	68.93	0	4H	0.29
SCWPU-10	65.67	0	4H	0.33
SCWPU-11	50.93	0	4H	0.35

**Table 5:** Film properties of coatings with different FAS−SiO_2_ contents.

Samples	Glossiness (60 °C)	Adhesion	Pencil Hardness
SCWPU/FAS−SiO_2_—10%	35.33	0	4H
SCWPU/FAS−SiO_2_—20%	23.75	0	4H
SCWPU/FAS−SiO_2_—30%	10.49	0	4H
SCWPU/FAS−SiO_2_—40%	5.90	1	5H
SCWPU/FAS−SiO_2_—50%	2.61	2	5H

## Data Availability

Data is contained within the article.
